# Barriers and facilitators to anal cancer screening among men who have sex with men: a systematic review with narrative synthesis

**DOI:** 10.1186/s12885-025-13980-w

**Published:** 2025-04-01

**Authors:** IatTou Sam, Wen Dang, NgaTeng Iu, ZiYue Luo, Yu-Tao Xiang, Robert David Smith

**Affiliations:** 1 Unit of Psychiatry, Department of Public Health and Medicinal Administration, & Institute of Translational Medicine, Faculty of Health Sciences, University of Macau, Macao, SAR China; 2 Centre for Cognitive and Brain Sciences, University of Macau, Macao, SAR China

**Keywords:** Barrier, Facilitator, MSM, Anal cancer, Screening

## Abstract

**Background:**

Increasing trends of anal cancer among men who have sex with men (MSM) highlight the importance of anal cancer screening. However, the screening rate of anal cancer among MSM remains relatively low. This systematic review aims to identify and critically evaluate studies examining barriers and facilitators influencing MSM’s participation in anal cancer screening.

**Methods:**

Systematic searches were performed in five databases (Web of Science, Medline, Embase, PsycINFO, and CINAHL). Evidence from qualitative, quantitative, and mixed methods studies was extracted and synthesized. Mixed Methods Appraisal Tool (MMAT) was used for quality assessment. Two researchers underwent selection and appraisal independently. PROSPERO registration number: CRD42024601449.

**Results:**

305 studies were identified with a total of 32 studies included, including 11 qualitative studies, 18 quantitative studies, and 3 mixed methods studies. The barriers and facilitators to anal cancer screening were categorized into four domains: individual factors, healthcare system factors, healthcare provider factors, and screen-related factors. Among the four domains, the most frequently reported barriers and facilitators to anal cancer screening were individual factors. A lack of knowledge about the risks of HPV, anal cancer, and anal screening (*n* = 16) was the most significant barrier. In contrast, a greater perceived understanding of anal cancer and screening (*n* = 6) was identified as the primary facilitator.

**Conclusions:**

This systematic review provided a comprehensive assessment of barriers and facilitators to anal cancer screening among MSM, highlighting the need for targeted comprehensive intervention programs to enhance acceptance of screening. Implementing effective strategies to address potential barriers and promote facilitators across all domains of public health could significantly increase screening uptake.

**Supplementary Information:**

The online version contains supplementary material available at 10.1186/s12885-025-13980-w.

## Background

Anal cancer accounts for less than 1% of all new cancer diagnoses, and the most common histological subtype is squamous cell carcinoma of the anus (SCCA) [1]. Despite being a relatively uncommon malignancy, the global burden of SCCA showed an increasing trend, with a rise from approximately 29,000 SCCA cases in 2018 [2] to over 30,000 cases in 2020 [3] reported globally. Early signs of cancer are thought to be high-grade squamous intraepithelial lesions (HSIL) that are assumed to be precursors of anal cancer [4–6]. The most common cause of anal cancers is attributed to prior infection of human papillomavirus (HPV), particularly the HPV16 variant [7]. The 5-year survival rate for people with anal cancer ranges from 63 to 86% [8–11]. Anal cancer imposes severe impacts on the quality of life, leading to distressing symptoms such as pain and bleeding [12]. Patients may need to live with a stoma when removal of the anal canal is required [13].

Men who have sex with men (MSM) are considered as people at increased risk for this disease, especially those living with human immunodeficiency virus (HIV) [14]. Risk behaviours such as condomless anal sexual intercourse and having a high number of different sexual partners may increase the risk of persistent HPV infection leading to a higher risk of developing anal cancer [1]. The prevalence of anal HPV and anal cancer precursors is high among MSM. A meta-analysis of 53 studies estimated the pooled prevalence of anal HPV-16 and HSIL of 13%, 29%, respectively, in HIV-negative MSM and 35%, 22% in MSM living with HIV [15]. This population represents 10 times increased risk of developing anal cancer compared to the general population [16]. This risk appears to increase with age as the reported incidence of anal cancer increases with age in MSM [17].

To address the increased risk of anal cancer among MSM populations, successfully implemented screening may provide an effective strategy for reversing this trend. Anal cytology, high-risk human papillomavirus (hrHPV) testing, hrHPV-cytology co-testing, digital anal rectal exam (DARE), and high-resolution anoscopy (HRA) are currently used methods for anal cancer screening and diagnosis [18]. Active monitoring and treating precancerous growths allow early detection and substantially prevent progression into anal cancer. A large multisite randomized controlled trial in the United States demonstrated the efficacy of detection and treatment of anal HSIL in people living with HIV [19]. The result from an observational study data in Australia showed anal cancer incidence was estimated to decline by 44–70% following the implementation of annual HSIL screening and treatment [20]. In 2024, the International Anal Neoplasia Society (IANS) reached an evidence-based consensus recommending the initiation of anal cancer screening at the age 35 years for MSM living with HIV and at 45 years for those not living with HIV [18].

While the effectiveness of screening in detecting anal cancer has been demonstrated in a randomized trial [19], the limited evidence available may hinder the uptake and adherence to anal cancer screening among MSM [21]. Surveys found only about 14% of the MSM individuals had received anal cancer screening [22], while the screening rates for other cancer types; prostate cancer and breast cancer are much higher, 37.1% and 75.9% respectively [23]. The relatively low rate of anal cancer screening among MSM indicates the need to address the screening uptake in terms of factors that are barriers and facilitators to screening in this population. Understanding the factors that influence screening decisions is crucial for successful implementation interventions aimed at increasing screening rates in people at increased risk [24]. By gaining insight into these factors, targeted screening interventions can be developed to encourage healthy behaviors and improve overall health outcomes within this people at increased risk.

Previous reviews have summarized barriers and facilitators related to various types of cancer screening in the general population [25] and sexual minority groups [26]. However, there are no systematic reviews identified that focused specifically on anal cancer screening among MSM. Given the burden of anal cancer in this people at increased risk and the potential for screening to detect this disease, a systematic review was conducted to identify and summarize studies examining the barriers and facilitators that influence MSM’s decision to participate in anal cancer screening. This review aims to provide a comprehensive evaluation of the factors that impact screening uptake in this population and to inform the development of interventions aimed at increasing screening rates.

## Methods

This systematic review is reported following the Preferred Reporting Items for Systematic Reviews and Meta-analyses (PRISMA) framework [27]. The study protocol was registered in the PROSPERO (Registration number: CRD42024601449).

A systematic literature search was conducted in five international databases (Web of Science, Medline, Embase, PsycINFO, and CINAHL) for relevant articles published between January 2008 and October 2023. The justification for the 2008 cut-off were based on two rationales. First, the European AIDS Clinical Society Guidelines published in 2008 recommend anal cancer screening for MSM living with HIV [28]. Second, we conducted a pilot search before 2008 and found the related literature was limited. Therefore, the reviewers decided by consensus that articles before 2008 were not sufficiently relevant to the current situation of the MSM population and chose the 2008 cut-off on the systematic search. Manual search of the bibliographies of previously published systematic reviews for any articles that may have been missed in the original search was conducted. The search terms were developed to capture the key concepts of “MSM”, “anus cancer screening”, and “barriers and facilitators” to retrieve all relevant studies. Detailed search terms were listed in the supplement file Table S1. Two researchers screened each retrieved study independently, firstly screening the title and abstract and then full-text screening. Inclusion criteria were set as qualitative/quantitative or mixed method studies with the identifying or evaluating the barriers and facilitators of anal cancer screening in MSM populations. Reviews, comments, editorials, conference papers, and case report papers were excluded. Publications not in English were excluded. Any disagreements were resolved through discussion and consensus.

Data extraction was performed by two independent researchers. Detailed information of each study was extracted to a standardized table including the author details, publication year, country, study type and design, participant characteristics, findings of the article, and the barriers and facilitators related to anal cancer screening identified from evidence in the studies. Once complete data extraction, two researchers compared each item and solved any discrepancy through consensus.

Due to heterogeneity across studies in terms of methods, sampling, design and measures data synthesis for barriers and facilitators related to anal cancer screening was done by a narrative synthesis based on a framework related to barriers and facilitators to health screening [25]. The themes from the framework were initially pilot-tested and subsequently enhanced with newly generated themes as they were identified within studies. Barriers and facilitators identified in the included studies were then organized and tabulated based on the final framework of themes. To prevent data from being improperly categorized, discrepancies were solved by further discussion with a senior researcher.

The Mixed Methods Appraisal Tool (MMAT) is a critical appraisal tool that is designed for the appraisal stage of systematic mixed studies reviews [29]. The evaluation process consists of three steps. First, two screening questions will be posed to determine whether the study qualifies as an empirical study. Responses can be “Yes”, “No” or “Can’t tell” with a response of “No” or “Can’t tell” to either question indicating that the paper is not an empirical study and therefore cannot be assessed using the MMAT. Second, the appropriate category of studies will be selected to appraise each included study. Third, the included study will be rated according to the methodology quality criteria of the chosen study category. The MMAT can assess the methodological quality of five categories of study: qualitative studies, quantitative randomized controlled trials, quantitative non-randomized studies, quantitative descriptive studies, and mixed methods research. Each study category includes 5 assessment criteria, with each criteria rating “Yes”, “No”, or “Can’t tell”. Studies did not report appropriate information to answer “Yes” or “No”, or that reported unclear information related to the criterion was rated as “Can’t tell”. Each criterion that receives a “Yes” earns one star, representing 20% of the total quality appraisal criteria met. In contrast, the highest quality studies achieve a total of five stars, accounting for 100% of the quality appraisal criteria met. Two researchers independently completed a quality assessment and then discussed and compared the scoring of each article until a consensus was agreed.

## Results

### Basic characteristics of included studies

**Fig. 1:**
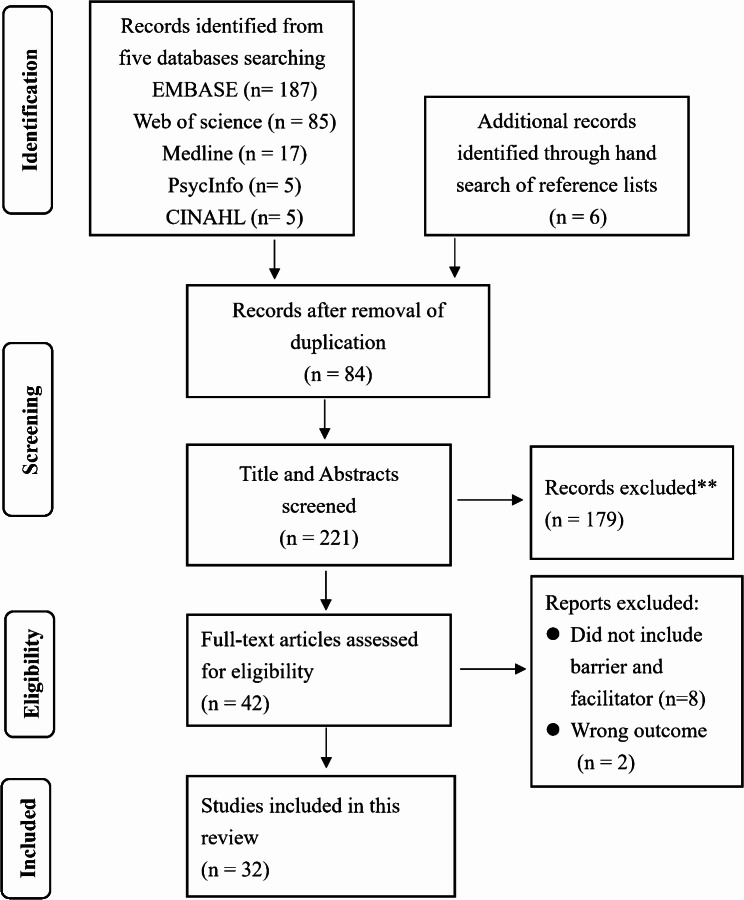
PRISMA flow diagram of the literature research

A total of 299 articles were identified from the five databases, and 6 additional records were identified through hand searches of reference lists of systematic reviews. After the exclusion of duplicates, 221 articles were screened in their title and abstracts. After the final full-text review, 32 papers fulfilled the inclusion criteria and were selected [21, 22, 30–59]. Figure 1 shows the PRISMA flow diagram of the literature research.

From the perspective of study design, 11 (34%) papers were qualitative studies [35, 38, 40, 41, 44, 46, 48–51, 58], 18 (56%) were quantitative studies [21, 22, 30–33, 36, 37, 39, 42, 45, 47, 52, 54–57, 59] and 3 (9%) were mixed methods studies [34, 43, 53]. Among 18 quantitative studies, 15 (83%) were quantitative non-randomized studies [21, 22, 30–32, 36, 37, 39, 45, 47, 52, 54–57], 2 (11%) were quantitative randomized controlled trials [33, 59], and 1 (6%) was quantitative descriptive studies [43]. About study location, 20 (63%) studies were conducted in the United States [21, 22, 31–33, 36, 39–45, 48–50, 54–56, 59], 4 (13%) from Australia [34, 35, 38, 46], 2 (6%) from Canada [37, 52], 2 (6%) from Pakistan [51, 58], and the United Kingdom [53], Puerto Rico [57], Thailand [30] each have one (3%) study. 1 (3%) study was conducted via online survey without focusing on a particular location [47]. For the study target population, more than half studies (78%) were focused on MSM and other sexual minority population perceptions [21, 22, 30–33, 35–37, 39–41, 43–49, 51, 52, 54, 55, 57, 59], other 7 (22%) studies were focused on healthcare providers and related workers’ perceptions [34, 38, 42, 50, 53, 56, 58]. The detailed characteristics of the included studies are shown in Table S2 in the supplementary file.

### Barriers and facilitators of anal cancer screening in MSM

Out of 32 studies, 25 (78%) reported facilitators, 27 (84%) barriers, 20 (63%) both barriers and facilitators. Barriers and facilitators to anal cancer screening emerged in four categories: individual factors, healthcare system factors, healthcare providers factors, and screen-related factors, with detailed information listed in Table 1. Among the four categories, the individual factors are the most reported factors influencing the uptake of anal cancer screening in MSM. 11 individual related factors were identified, including age, ethnicity, sexual orientation, education, health condition, health behavior, health literacy, attitude, psychology, resources, and social support. Factors within the health system included efficiency of the system, dissemination of the information as well as financial support. Healthcare providers’ factors included professionalism, attitude, and relationship with patients. Screen-related factors are related to the screening itself, such as screening method, screening environment, cost, and convenience for accessing screening.

**Table 1:** Barriers and facilitators to anal cancer screening in MSM synthesized from all studies

Factors	Facilitators	Barriers
Individual factors		
**Age**		Younger age [32]
**Race**	Black race [21]	Non-Hispanic Black race [32] Racialized groups [52]
**Sexual orientation**	Homosexuals [59]	Heterosexual men [52], bisexual or queer [59]
**Resource**	Availability of the screening test [21, 54, 57] Education from a health care provider or accept education program [36, 54] Having insurance coverage [21] Having a high annual household income [22]	Financial distress [30, 32, 42, 47, 51, 55, 57] Lack of availability of the screening test [39, 56] Lack of time for participating due to work, traffic etc. [43, 57]
**Education**	More years of school [59]	Not having a college degree [32]
**Health condition**	Living with HIV[21, 22, 39, 51] Self-observed, other perceived or diagnosed anal disease or physical symptoms [21, 30, 31, 45, 57] Family history of cancer[57]	Living with HIV [59] Not experiencing anal cancer symptoms [57] Mental health conditions [42]
**Health behavior**	Previous history of anal cancer screening [21, 22] Higher levels of sexual activity (i.e., anal receptive sexual partners) in the previous six months [21, 31]	Not disclosed their sexual behavior with men to their primary health care provider [22] Not having anal receptive sexual intercourse [32]
**Health literacy**	Greater perceived knowledge about anal cancer screening, anal cancer, HPV, vaccination and associated risk [22, 31, 37, 39, 43, 46, 57]	Lack of knowledge related HPV, anal cancer, and anal screening [32, 35, 39–45, 47, 49, 50, 55–58] Lack of awareness and concern of disease risk [35, 38–40, 42, 45, 49, 56, 57] Uncertainty about the effectiveness and possible side effect of anal cancer screening [32, 48, 52] Uncertain about their doctor’s recommendation [52]
**Psychology**	Worry about getting anal cancer and being very concerned about anal cancer [21, 22, 39, 43] Less anal sex stigma [54] High tolerance for any pain experienced [43]	Stigma and discrimination [39, 41, 43, 44, 49–51, 54, 58] Psychological distress, such as anxious/fear about anal cancer screening and the finding an abnormality [32, 35, 40, 43, 44, 48, 55, 57] Feel shame or embarrassment [39, 41, 43–45, 47, 50, 57] Negative experience [47, 48] Having other health concern (i.e., living with HIV) [40] Vulnerability [48]
**Attitude**	Patient willingness [44, 45, 50, 56, 57] Perceptions of self-efficacy [41, 43, 52] Safer sex fatigue [21] Reduced HIV concern [21] Not believing anal Pap tests are only necessary for people who have anal intercourse [22] Positive normative beliefs [52] Positive behavioral beliefs regarding treatment [52] Belief about HPV-related disease or HRA [43]	Not Interested in or dislike anal screening and potentially toughing faeces [32, 45, 54, 57] Sensitive nature of discussing sexual identity and sexual practices and screening itself [35, 48] Self-esteem, male affect their masculinity [44] Internalized racism [48] Connection the HRA to the Sexual Behavior “Bottom” Identity [49]
**Social support**	Recommended by the healthcare provider [21, 44, 56, 57] Good communication and relationship with the healthcare provider [37, 43, 46, 48, 56] Good communication with supportive sexual partner [50] Close relationship experiences the disease of prostate cancer [49] Discussion with female friend [49] Social support [54]	Lack of recommendation from healthcare professional [40, 47, 57]
**Healthcare system factors**		
**Efficiency of the system**	Improving health check-up processes [31, 50, 51, 55] Development of clear screening guideline [56, 58]	Healthcare system inefficiencies, such as the absence of a clinical routine, unclear delineation of responsibilities, weak health information system, health service not meet the specific need, ambiguous policy guidelines [38, 43, 48, 54, 58] Differences in HIV care practices [38]
**Resources**	Training healthcare providers [40, 51, 56, 58]	Lack of human resources [34, 41–43, 58] Inadequate infrastructure [53, 58] Lack of funding and financial resources [38, 58] Lack of political will and commitment from the government [58]
**Dissemination**	Creation of dissemination style on health education [40]	No promotion in media [34]
**Healthcare provider factors**		
**Professional**	Well trained healthcare provider with adequate knowledge, expertise and good communication skill [43, 44, 48, 50]	Concern about the effectiveness and acceptability of anal screening [34, 38, 53] Lack of professional training [53] Lack of time [34, 42]
**Attitude**	Non-judgment [48]	Bias, apathy and discourtesy from the doctor’s side [48, 51] Forgetting (i.e., lack of reminders) [34] Unnecessary anxiety, discomfort, embarrassment for patients [34] Low patient interest [34]
**Relationship**	Good communication and relationship with the patient [43, 48, 56]	Poor communication with the patient [38, 43]
**Screen-related factors**		
**Screening method**	Self-anal exams [44, 45] Preferred anal examinations to be performed only by a medical expert [35] Partner anal exams [45] Self-anal examination can be taught by the clinician [41]	Discomfort feelings from anal cancer screening [32, 33, 35, 38, 39, 43, 50, 54, 57] Inadequate physical flexibility to conduct an anal self-examination [35, 45] Concerned about hygiene before anal self-examination [35]
**Screening environment**	Screening performs in familiar environment [59] Friendly environment [48]	Unfriendly therapeutic environment [50]
**Cost**		Cost of screening [39, 57] Time consuming [39]
**Convenience**	Convenience of the screening method [44]	Lack of convenience of the screening [39]

The most reported individual factor barriers to anal cancer screening were lack of knowledge related to HPV, anal cancer, and anal screening (*n* = 16). Healthcare system inefficiencies (*n* = 5) and lack of human sources (*n* = 5) were the most reported healthcare system barriers for MSM to uptake anal cancer screening. For healthcare providers, concerns about the effectiveness and acceptability of anal screening (*n* = 3) were the most common barrier. For the screening-related factors, the discomfort feelings from anal cancer screening (*n* = 9) and inadequate physical flexibility to conduct an anal self-examination (*n* = 2) affect MSM’s decisions on the uptake of the appropriate screening.

The most reported facilitator to anal cancer screening on individual factors was greater perceived knowledge about anal cancer and screening (*n* = 7). For healthcare systems, improving health check-up processes (*n* = 4) and training healthcare providers (*n* = 4) were the top two reasons for facilitating. For healthcare providers, well-trained healthcare providers with adequate knowledge, expertise, and good communication skill (*n* = 4) seemed to be the most common factor increasing MSM’s attitudes to anal cancer screening. Having the practical ability to conduct anal examinations by themselves (*n* = 2) also facilitates MSM’s acceptance of anal cancer screening.

### Individual factors

Individual factors contributed to the barriers and facilitators for MSM receiving anal cancer screening. A consistent and prominent barrier was a lack of knowledge among participants [32, 35, 39–45, 47, 49, 50, 55–58]. Around 50% of this risk population understood that HPV was associated with anal cancer [55], while some individuals even had never heard of anal cancer or the need to screen for this disease [40, 44]. Knowledge gaps exist in a wide range of domains, such as HPV, HIV, anal cancer, and anal cancer-related risk factors. Insufficient knowledge may result in misconceptions about personal risk and a lack of awareness of risk factors. For instance, the categorization of MSM leads some individuals to believe that only the receptive partner in anal intercourse is at risk for this disease, while the incentive partner is not [32, 35, 49]. The belief in self-education raises their desire for the healthcare provider to share as much knowledge with them [48]. However, compared to men having sex with women, MSM demonstrated greater knowledge regarding anal screening [57]. MSM not living with HIV had lower HPV knowledge scores than those who living with HIV [39], highlighting the need for relevant interventions in specific populations in MSM.

Several psychological factors, such as stigma [39, 41, 43, 44, 49–51, 54, 58], psychologic distress [32, 35, 40, 43, 44, 48, 55, 57], and embarrassment [39, 41, 43–45, 47, 50, 57] experienced by MSM, were found to impede health-seeking behaviors. Experienced stigma, along with the lack of sex education and information, contributed to internalized stigma and anticipation of future devaluation, which further inhibited health-seeking behavior [50]. Individuals who endorsed less anal sex stigma were more likely to have ever received anal health screening by a physician [54]. Most of the psychologic distress stemmed from the worries about the abnormality resulting from anal screening [35, 40, 43, 44], in contrast, worries about developing anal cancer may motivate them to seek health checks [21, 22, 39, 43]. Although two cross-sectional studies found that MSM with a previous history of anal cancer screening showed a higher acceptance of screening [21, 22], people with negative experience of screening might be deterred from pursuing further anal cancer screenings [47, 48], while individuals with high pain tolerance experienced showed higher acceptance [43].

Individual attitudes and self-sexual identity also played significant roles. Those exhibited positive beliefs and greater willingness for healthcare-seeking behaviors [44, 45, 50, 52, 56, 57] and high self-efficacy [41, 43, 52]showing higher acceptance of anal cancer screening. Cross-sectional studies conducted by D’Souza et al. and Reed et al. also suggested individuals with attitudes such as safer sex fatigue and reduced concern about HIV, and the belief that anal screening is only necessary for people who have anal intercourse were more likely to engage in anal cancer screening [21, 22]. However, people’s low interest or dislike of screening was the other reason for rejecting screening, particularly their concerns about potentially touching feces during self-anal examinations [32, 45, 54, 57]. Self-disclosure of sexual experience may be sensitive for MSM, which can inhibit their willingness to seek help from healthcare providers [35, 48]. Additionally, the idea of linking anal cancer screening to the ‘bottom’ identity in sexual behavior [49] and concerns about losing masculinity [44] may hinder MSM from undergoing anal screening.

Valid social support from close relationships, sexual partners, and healthcare providers may improve the acceptance of screening [37, 43, 46, 48–50, 54, 56]. Greater social support is associated with lower levels of internalized stigma among MSM, which is a barrier to anal cancer screening [48]. Most studies agreed on the positive impact of healthcare providers’ social support [21, 44, 56, 57]. Effective communication and recommendations for anal screening instill confidence in patients, encouraging them to pursue necessary health checks. In contrast, a lack of recommendation from healthcare professionals may undermine their confidence in accepting anal cancer screening [40, 47, 57].

Other individual factors, such as race and ethnicity [21, 32, 52], sexual orientation [52, 59], education level [32, 59], individual financial status [21, 22, 30, 32, 42, 47, 51, 55, 57], availability of the screening test [21, 39, 54, 56, 57], and health conditions [21, 22, 30, 31, 39, 42, 51, 57, 59] were found associated with the screening decision. The role of ethnicity appeared both as a positive and negative correlate with screening participation [21, 32, 52]. Additionally, compared to those experiencing anal cancer symptoms [57], individuals who had self-identified or been diagnosed with anal diseases or physical symptoms showed a strong desire to get screened [21, 30, 31, 45]. People living with HIV were found less likely to engage in screening [59], while positive HIV status motivates MSM to get anal cancer screening [21, 22, 39, 51].

### Healthcare system factors

In the healthcare system domain, issues like healthcare system inefficiencies [38, 43, 48, 54, 58], lack of human resources [34, 41–43, 58], inadequate infrastructure [53, 58], lack of financial support from the government [38, 58], and lack of different forms of dissemination [34, 40] further compounded these systemic challenges.

The efficiency of healthcare systems was regarded as a significant factor influencing the intention to seek health checks and adaption of anal screening. From the perspective of clinicians, specialist physicians, or other key informants, the inefficiency of the healthcare system is mainly reflected in the absence of a clinical routine, unclear delineation of responsibilities, weak health information system, and unmet service needs [38, 58]. The absence of a clinical routine and unclear delineation of responsibilities can lead to clinical physician confusion about who is responsible for patient referrals, resulting in delays in screening. Similarly, a weak health information system may not be able to track the patient’s eligibility for regularly monitoring, while unmet service needs can prevent patients from accessing necessary healthcare resources. From the perspective of MSM, the lack of clear clinical and policy guidelines, along with systemic inefficiencies related to scheduling, negatively influences their intention to seek screening and engage in follow-up checks [43, 48, 54]. Improving the processes of health checks [31, 50, 51, 55] and developing related guidelines [56, 58] were considered to be a possible way of facilitating the uptake of anal screening.

The shortage of human and infrastructure resources and a lack of financial incentives underscores the importance and urgency of the rational allocation of human, material, and financial resources. Four studies highlighted the need for training professional healthcare providers [40, 51, 56, 58]. A strong leadership commitment was a key to overcoming the barriers [58]. Additionally, we found that physicians from Australia claimed that no promotion in media was one of the barriers to screening [34], indicating the positive role of disseminating information. Policymakers and healthcare organizations should take action to address inefficiencies, train healthcare providers, improve infrastructure, provide adequate financial support, and ensure dissemination of information about the importance of anal cancer screening.

### Healthcare providers factors

Findings highlight the influential role that healthcare providers can play in encouraging and supporting screening participation among this population. Well-trained professional health providers with adequate knowledge, expertise, and good communication skills can facilitate patients’ acceptance of anal examinations [43, 44, 48, 50]. However, the lack of professional training and insufficient work time for conducting these examinations pose challenges to their ability to offer comprehensive and high-quality services [34, 42, 53]. Healthcare providers expressed their concern about the effectiveness and acceptability of anal screening which negatively influenced the screening participants [34, 38, 53]. This finding highlights the necessity for high-quality evidence to enhance the confidence of both healthcare providers and MSMs.

Our study also identified that physicians’ attitudes toward patients can influence their health-related behaviors. A non-judgmental attitude from physicians towards MSM was a necessary factor in building trust and facilitating screening uptake, while bias, apathy, and discourtesy may be barriers to screening decisions [34, 48, 51]. Additionally, having good communication and relationship with patients were generally perceived as a facilitating factor of anal screening and follow-up checks [43, 48, 56]. But a small number of physicians expressed difficulties in initiating the topic of anal cancer screening with patients [38].

### Screen-related factors

Apart from individual factors, healthcare systems, and healthcare provider-related factors, the practice of anal screening itself also impacts patients’ decisions, such as screening method [32, 33, 35, 38, 39, 41, 43–45, 50, 54], screening environment [48, 50, 59], cost [39, 57], and convenience of the screening method [39, 44].

Discomfort feelings caused by the invasive nature of screening procedures such as HRA affected the screening decision [32, 33, 35, 38, 39, 43, 50, 54, 57]. However, those with a higher pain tolerance were more confident in receiving screening [43]. Some MSMs prefer anal examinations to be performed exclusively by health professionals [35], and others think partner-administered anal examinations are acceptable as well [45]. However, most MSMs still prefer self-anal screening with clear procedure [44, 45], as greater safety and privacy. This preference stemmed from a need to feel in control of the screening process and avoid potential discomfort or embarrassment associated with provider-administered screening procedures. We also identified that self-anal examination taught by healthcare professionals was more acceptable to MSM [41]. This suggests that empowering MSM with the knowledge and skills to perform self-screening, under the guidance of trained providers, may be a more acceptable approach for MSM than solely relying on healthcare providers. However, Concerns about hygiene before the self-examination and a lack of physical flexibility for some people also play as a barrier [35, 45].

Additionally, screening performed in a familiar environment or friendly environment can facilitate MSMs’ acceptance of anal screening [48, 50, 59]. Financial and time-related costs associated with screening are also significant practical factors influencing the decision-making of MSM [39, 57]. Furthermore, individuals are concerned about the convenience of the screening methods available [39, 44].

### Quality assessment and risk bias of included studies

The included studies displayed a broad range of quality, from 20 to 100%. Most studies met either 100% (13 studies, 41%) or 80% (9 studies, 28%) of all quality assessment criteria. 7 studies (22%) met 60% of the criteria, 1 (3%) study met 40%, and 2 (6%) studies met 20% of the quality assessment criteria. In general, the included qualitative studies satisfied most of the criteria but revealed significant variability in quantitative and mixed methods studies. 9 out of 11 qualitative studies met all the criteria, except Russo et al. and Koskan et al. achieving 80% and 60% of the criteria respectively. The quality of quantitative studies displayed a range from 60 to 100%, as only a few studies satisfied criteria 2 (sample representative) and criteria 4 (response rate) and lower-rated studies often lacked clarity and completeness as indicated by multiple “Can’t tell” responses. Meanwhile, the mixed methods studies displayed relatively lower quality than qualitative and quantitative studies. The quantitative component is rated high quality while the qualitative component is rated low quality, the overall rating for this criterion will be of low quality. Table S3 in the supplementary file shows the detailed result of the quality assessment using MMAT.

## Discussion

This systematic review provided insights into the understanding of barriers and facilitators to anal cancer screening in MSM. It identified 51 barriers and 45 facilitators to MSM individuals accessing anal cancer screening from 32 studies. The findings suggest that a range of factors including individual factors, healthcare system factors, healthcare providers factors, and screen-related factors are contributors to the barriers and facilitators for MSM receiving anal cancer screening.

The barriers and facilitators identified can provide clear direction for improvement among MSM. The most common individual barriers to anal cancer screening were a lack of knowledge about anal cancer and related risk factors, screening itself, a lack of awareness of disease risk, and perceived stigma and discrimination. Healthcare system barriers are related to inefficiency of the system, lack of human resources, lack of financial support, and lack of dissemination. For healthcare providers, the barriers are related to the lack of professional knowledge and training, attitude towards MSM, and relationship with MSM. At the screening-related level, participants noted the discomfort experienced by screening, concerns about the environment and cost, and lack of convenience of the screening.

Demographic factors related to barriers and facilitators to anal cancer screening were reported in a few studies and these results may help guide further researchers to identify the target population among sexual minorities. Other modifiable individual factors, such as a lack of knowledge, a lack of awareness of disease risk, and perceived stigma and discrimination may be addressed by improving targeted interventions [60]. Intervention elements with the topic of stigma and discrimination should be taken into consideration when developing multilevel interventions for MSM. Gunn et al. conducted a systematic review and meta-analysis that suggested a stigma reduction component in interventions can improve HIV test and reduce sex risk for MSM [61]. By enhancing awareness and reducing societal stigma, we can create a more positive environment for MSM to engage in discussions about their health [36, 48, 56].

This review also found that well-trained healthcare providers positively influence participants’ willingness to undergo screening, providing healthcare providers with professional training is essential. Barriers like providers lack of professional knowledge about the screening guideline and lack of standardized screening protocols significantly affect the rate of anal cancer screening [34, 52]. A streamlined clinical process and enhanced training for healthcare providers to increase the awareness of the participants can significantly improve the screening rates [31, 55]. It highlights the need for a well-designed training program that should encompass knowledge related to anal cancer and screening, clinical practice, and the development of communication skills.

However, there was limited evidence to demonstrate the effect of anal cancer screening for MSM. Only one randomized controlled trial demonstrated the HRA for detection and treatment has a 57% reduction of progression to anal cancer [19]. Without sufficient high-quality evidence of the effect of anal screening for MSMs healthcare providers may raise concerns about the true effectiveness and side effects of anal cancer screening. The recently published international clinical consensus guideline for anal cancer screening in people at increased risk may enhance healthcare providers’ confidence when engaging in discussions with patients [18]. In the future, more high-quality research is still needed to provide robust evidence for both healthcare providers and patients.

With the continuous advancement of anal cancer screening technologies, selecting appropriate screening tools is crucial for improving detection efficiency and reducing the waste of medical resources. Given the socioeconomic barriers to accessing healthcare and the inherent limitation of anal screening infrastructure, anal self-exam becomes a valuable screening method for anal cancer in MSMs [62]. Recently, Nyitray and colleagues assessed the accuracy of individuals to self-detect smaller anal abnormalities [63]. The research suggested the sensitivity and specificity of the self-exams were 59.6% and 80.2%, respectively, and the overall concordance between self-exams and DARE increased with increasing anal canal lesion size. Results from a systematic review showed that sample adequacy for cytology testing was about 10% lower by self-collection than clinician-collection [64]. Self-exams of the anal region can serve as an alternative approach for early detection of invasive tumors, particularly in underserved rural areas. Therefore, development and optimization of educational tools and awareness strategies will be essential to the widespread adoption of this method.

### Comparison to other studies

The barriers and facilitators for anal cancer screening among MSM can also be evidence for improving other healthcare behaviors. Our findings regarding barriers and facilitators to anal screening align with those identified in the context of HIV test, including factors such as lack of knowledge and stigma [65, 66]. Given the similarities in these identified factors, a systematic approach to addressing them may benefit not only anal cancer screening but also other health conditions and risks within this population. Furthermore, many of the barriers addressed in this study can apply universally to many organized screening initiatives, such as fear of pain/discomfort, stigma, disgust (e.g., relevant to colorectal cancer screening) although anal cancer in MSM is a unique subgroup who also experience complex and sometimes challenging interactions with the health system due to discrimination.

### Implications for clinical practice and research

Based on the findings of this review, some points were identified to inform the health profession and the public when addressing cancer screening for MSM. First, provision and access to comprehensive education and training programs to empower MSM with the knowledge and skills for anal cancer and screening. Innovative forms of communication contribute to improving relevant knowledge of the target population [67]. Public health may also consider public awareness campaigns to improve understanding of anal cancer and screening practices. Second, the topic of stigma and discrimination should be taken into consideration when developing multilevel interventions for MSM [61]. Third, training healthcare professionals to improve disease-related knowledge and cultural competencies regarding the health needs of MSM to provide care in a manner free of discrimination. Training courses, such as Health4LGBTI, increased knowledge of lesbian, gay, bisexual, trans and intersex (LGBTI) health and improved attitudes toward LGBTI people [68].

Future research should focus on developing systematic intervention programs to address these modifiable barriers and facilitators of anal cancer screening among MSM. Evaluate the effectiveness of these programs to provide robust evidence for their implementation across various regions. Additionally, considering the accessibility and value of anal self-exams, it is crucial to evaluate their long-term effectiveness further and explore applicability in low-resource areas.

### Strengths and limitation

The study has the following advantages. This review provides a nuanced understanding of the barriers and facilitators associated with anal cancer screening among MSM by synthesis of quantitative, qualitative, and mixed methods studies. It allowed us to capture factors influencing anal cancer screening participation and help inform the development of future interventions and research to promote effective anal cancer screening practices. Independent reviewing process and quality assessment of studies with different study designs ensures the validity and reliability of the findings.

However, our review still has several limitations. Firstly, although a broad search strategy was employed to address the research question, some articles may have been overlooked due to the lack of specific search terms. Secondly, the small sample size of some studies included in this review may compromise the accuracy of the findings. Finally, all studies were in English and were conducted in Western countries. While many perspectives from these studies are likely applicable to other countries and regions, the review would be more representative if a diverse range of articles from different regions were included.

## Conclusion

This systematic review provides insight into understanding multifaceted factors affecting screening uptake among MSM. Lack of knowledge of anal cancer and the relative risk was perceived as the most important factor affecting the screening uptake, beside the barriers are also contributed by psychological factors and the screening procedure, such as stigma and discomfort during the screening. Conversely, the intention of the screening uptake is not only contributed by individual factors, but also influenced by the healthcare system, healthcare providers, and screen-related factors. Effective strategies to eliminate the possible barriers and promote facilitators within all domains of public health may contribute to increase uptake in screening.

## Electronic supplementary material

Below is the link to the electronic supplementary material.

Supplementary Material 1

## Data Availability

The authors confirm that the data supporting the findings of this study are available within the article and its supplementary materials.

## Abbreviations

AIDS: Acquired immunodeficiency syndrome
DARE: Digital rectal examination
IANS: International anal neoplasia society
LGBTI: Lesbian, gay, bisexual, trans and intersex
MSM: Men who have sex with men
MMAT: Mixed method appraisal tools
HRA: High resolution anoscopy
HSIL: High-grade squamous intraepithelial lesions
HPV: Human papillomavirus
HrHPV: High-risk human papillomavirus
HIV: Human immunodeficiency virus
SCCA: Squamous cell carcinoma of the anus
