# Supplementary material for: Barriers and facilitators to anal cancer screening among men who have sex with men: a systematic review with narrative synthesis
Source: BMC Cancer. 2025 Apr 1;25:586. doi: 10.1186/s12885-025-13980-w (PMC11963451; doi:10.1186/s12885-025-13980-w)
Supplement: Supplementary file 1 — Supplementary Material 1 [file 12885_2025_13980_MOESM1_ESM.docx]

Supplement file

[Table S1 Search term of each database 2](#_Toc181712999)

[Table S2 The detailed characteristics of the included studies 3](#_Toc181713000)

[Table S3 Result of the quality assessment using MMAT 6](#_Toc181713001)

[Reference 9](#_Toc181713002)

**Table S1 Search term of each database**

| Database | Search strategy |
| --- | --- |
| Web of Science | Ti or ab:( Facilitators OR barriers OR attitude OR behavior OR behaviours OR behave*) AND Ti or ab: (Anal OR anus OR ano* OR anal gland OR anal canal OR anal duct) AND Ti or ab: (cancer OR carcinoma OR neoplasm OR malignancy OR squamous cell carcinoma OR squamous cell cancer) AND Ti or ab: (Screening OR screen* OR diagnoses OR cancer screening OR assessment) AND Ti or ab: (“MSM” OR “MSMW” OR men who have sex with men and women OR men having sex with men OR Men who have sex with men OR gay OR bisexual OR not-exclusively heterosexual OR sex minority OR Homosexuality, Male OR same-sex) |
| PsyInfo | Ti or ab:( Facilitators OR barriers OR attitude OR behavior OR behaviours OR behave*) AND Ti or ab: (Anal OR anus OR ano* OR anal gland OR anal canal OR anal duct) AND Ti or ab: (cancer OR carcinoma OR neoplasm OR malignancy OR squamous cell carcinoma OR squamous cell cancer) AND Ti or ab: (Screening OR screen* OR diagnoses OR cancer screening OR assessment) AND Ti or ab: (“MSM” OR “MSMW” OR men who have sex with men and women OR men having sex with men OR Men who have sex with men OR gay OR bisexual OR not-exclusively heterosexual OR sex minority OR Homosexuality, Male OR same-sex) |
| Medline | tiab(Facilitators/Mh OR barriers/Mh OR attitude OR behavior OR behaviors OR behave*) AND tiab (Anal/Mh OR anus OR ano* OR anal gland OR anal canal OR anal duct) AND tiab (cancer/Mh OR carcinoma/Mh OR neoplasm OR malignancy OR squamous cell carcinoma OR squamous cell cancer) AND tiab (Screening/Mh OR screen* OR diagnoses OR cancer screening OR assessment) AND tiab (MSM OR MSMW OR men who have sex with men and women OR men having sex with men OR Men who have sex with men OR gay/Mh OR bisexual/Mh OR not-exclusively heterosexual OR sex minority OR Homosexuality, Male OR same-sex) |
| Embase | ab,sh,ti. ("facilitator*" OR "barrier*" OR attitude OR "behavior*" OR "behavior*" OR "behave*") AND.ab,sh,ti. (Anal OR Anus OR anal gland OR anal canal OR anal duct) AND.ab,sh,ti.(cancer OR carcinoma OR neoplasm OR malignancy OR squamous cell carcinoma OR squamous cell cancer) AND ab,sh,ti.(MSM OR MSMW OR men who have sex with men and women OR men having sex with men OR Men who have sex with men OR gay OR bisexual OR not-exclusively heterosexual OR sex minority OR Homosexuality OR same-sex) |
| CINAHL | (AB ( Facilitators OR barriers OR attitude OR behavior OR behaviors OR behave* ) OR SU (Facilitators OR barriers OR attitude OR behavior OR behaviors OR behave*)) AND (AB (Anal OR anus OR ano* OR anal gland OR anal canal OR anal duct ) OR SU ( Anal OR anus OR ano* OR anal gland OR anal canal OR anal duct)) AND (AB (carcinoma OR neoplasm OR malignancy OR squamous cell carcinoma OR squamous cell cancer ) OR SU (carcinoma OR neoplasm OR malignancy OR squamous cell carcinoma OR squamous cell cancer)) AND (AB (Screening OR screen* OR diagnoses OR cancer screening OR assessment ) OR SU (Screening OR screen*OR diagnoses OR cancer screening OR assessment)) AND (AB (“MSM” OR “MSMW” OR men who have sex with men and women OR men having sex with men OR Men who have sex with men OR gay OR bisexual OR not-exclusively heterosexual OR sex minority OR Homosexuality, Male OR same-sex ) OR SU (“MSM” OR “MSMW” OR men who have sex with men and women OR men having sex with men OR Men who have sex with men OR gay OR bisexual OR not-exclusively heterosexual OR sex minority OR Homosexuality, Male OR same-sex)) |

**Table S2 The detailed characteristics of the included studies**

| **Author** | **Year** | **Location** | **Study type** | **Study design** | **Sample size** | **Age (year)** |
| --- | --- | --- | --- | --- | --- | --- |
| Acree et al [1] | 2020 | USA | Qualitative study | Semi-structured interview and focus group discussion | 45 Black cisgender men | NR |
| Butame et al [2] | 2017 | USA | Qualitative study | Focus group discussion | 24 MSM | Mean: 54 Range: 31 - 76 |
| Ejaz et al [3] | 2022 | Pakistan | Qualitative study | Focus group discussions | 29 Homosexual, 9 bisexual, and 10 transgender women | Median (range):  22.5 (20 - 25) |
| Ejaz et al [4] | 2023 | Pakistan | Qualitative study | Semi-structured interview | 18 Key informants | NR |
| Finneran et al [5] | 2021 | USA | Qualitative study | Focus group discussion | 15 Sexual minority men (Homosexual/gay/Bisexual/unknow) | Mean: 51.2 Range: 44 - 67 |
| Koskan et al [6] | 2016 | USA | Qualitative study | In-depth interviews | 58 HIV-positive MSM | Above 21 |
| Koskan et al [7] | 2018 | USA | Qualitative study | In-depth interviews | 33 Foreign-born Latino HIV-infected GBM | Mean: 44 Range: 22 - 68 |
| Kutner et al [8] | 2021 | USA | Qualitative study | In-depth interviews | 10 Key informants and 25 MSM | KI: 44.6 (12.6) MSM: 34.0 (9.1) |
| Ong et al [9] | 2014 | Australia | Qualitative study | Semi-structured interview | 20 HIV-positive MSM | Mean: 54.5  Range: 35 - 78 |
| Ong et al [10] | 2015 | Australia | Qualitative study | Semi-structured interview | 20 HIV physicians | Mean: 51.6 Range: 35 - 61 |
| Russo et al [11] | 2018 | Australia | Qualitative study | In-depth interviews | 21 GBM/MSM | Above 35 |
| Davis et al [12] | 2013 | USA | Quantitative study | Randomized trial | 296 MSM | Mean: 43  Range: 21 - 79 |
| Nyitray et al [13] | 2023 | USA | Quantitative study | Randomized trial | 240 Cisgender sexual minority men and transgender people | Media (range):  46 (33 - 57) |
| Apaydin et al [14] | 2018b | USA | Quantitative study | Cross-sectional study | 33 primary care providers | Mean: 40 |
| Cruz et al [15] | 2023 | Puerto Rico | Quantitative study | Cross-sectional study | 202 People living with HIV (88 MSM, 48 MSW, 65 women, 1 unknow) | Median (range):  54 (46 - 58) |
| D' Souza et al [16] | 2013 | USA | Quantitative study | Cohort study | 1742 MSM | Median (range):  55 (49 - 61) |
| D'Souza et al [17] | 2008 | USA | Quantitative study | Cross-sectional study | 1917 MSM | Mean (SD): 48.3 (10.1) |
| Fenkl et al [18] | 2015 | USA | Quantitative study | Cross-sectional study | 94 HIV-positive MSM | Mean (SD): 51.9 (9.8) |
| Fenkl et al [19] | 2016 | USA | Quantitative study | Cross-sectional study | 163 MSM | Mean (SD): 49.6 (14.7) Range: 39 - 59 |
| Gillis et al [20] | 2022 | Canada | Quantitative study | Cross-sectional study | 1677 Men living with HIV (72% gay, 7% bisexual, 5% other men who have sex with men, 16% heterosexual) | Median (range):  53 (45–59) |
| Kutner et al [21] | 2022 | USA | Quantitative study | Cross-sectional study | 1513 Adult cisgender GBM | Mean (SD): 36.1 (11.3) Range: 18 - 72 |
| Li et al [22] | 2009 | Thailand | Quantitative study | Cross-sectional study | 174 MSM | Mean (SD): 32.1 (8.3) Range: 19 - 57 |
| Lombardo et al [23] | 2022 | USA | Quantitative study | Cross-sectional study | 422 individuals who self-identified with the LGBTQ+ community | Above 18 |
| Moores et al [24] | 2015 | Canada | Quantitative study | Cross-sectional study | 280 MSM | Mean (SD): 37 (11.86) Range: 18 - 69 |
| Nyitray et al [25] | 2018 | USA | Quantitative study | Phase 2 clinical feasibility study | 200 MSM | Median (range):  52 (27 - 78) |
| Olusanya et al [26] | 2022 | USA | Quantitative study | Cross-sectional study | 30 Employees/volunteers from three HIV/AIDS service organizations | Mean (SD): 46.5 (13.5) |
| Rahman et al [27] | 2019 | NR | Quantitative study | Cross-sectional study | 87 Ciswomen, 34 transwomen, and 27 transmen | Mean (SD): 27.1 (7.29) |
| Reed et al [28] | 2010 | USA | Quantitative study | Cross-sectional study | 236 Gay and 70 bisexual people | Mean (SD): 46.4 (9.0) Range: 18 - 59 |
| Truesdale et al [29] | 2010 | USA | Quantitative study | Cohort study | 195 MSM | Mean (SD): 43.6 (9.7) |
| Apaydin et al [30] | 2018a | USA | Mixed method study | Survey and focus groups interview | 44 MSM | Mean (SD): 49 (9.9) Range: 27 - 61 |
| Hughes et al [31] | 2022 | UK | Mixed method study | Survey and free text response | 25 Doctors and 15 nurses | NR |
| Ong et al [32] | 2013 | Australia | Mixed method study | Survey and free text response | 36 Physicians (8 sexual health physicians, 14 general practitioners, and 14 infectious disease physicians) | NR |

**Footnote:** NR: Not report; MSM: Men who have sex with men; GBM: Gay and bisexual men; KI: key informants; HIV: Human immunodeficiency virus; AIDS: Acquired immunodeficiency syndrome; LGBTQ: lesbian, gay, bisexual, transgender and queer or questioning.

**Table S3 Result of the quality assessment using MMAT**

| **Author and year** | **All Q1** | **All Q2** | **Q1** | **Q2** | **Q3** | **Q4** | **Q5** | **Quality** | |
| --- | --- | --- | --- | --- | --- | --- | --- | --- | --- |
|  | Are there clear research questions? | Do the collected data allow to address the research questions? | ***Qualitative study*** | | | | |  |  |
|  |  |  | Is the qualitative approach appropriate to answer the research question? | Are the qualitative data collection methods adequate to address the research question? | Are the findings adequately derived from the data? | Is the interpretation of results sufficiently substantiated by data? | Is there coherence between qualitative data sources, collection, analysis and interpretation? |  |  |
| Acree et al., 2020 [1] | Yes | Yes | Yes | Yes | Yes | Yes | Yes | 100% | ***** |
| Butame et al., 2017 [2] | Yes | Yes | Yes | Yes | Yes | Yes | Yes | 100% | ***** |
| Ejaz et al., 2022 [3] | Yes | Yes | Yes | Yes | Yes | Yes | Yes | 100% | ***** |
| Ejaz et al., 2023 [4] | Yes | Yes | Yes | Yes | Yes | Yes | Yes | 100% | **** |
| Finneran et al., 2021 [5] | Yes | Yes | Yes | Yes | Yes | Yes | Yes | 100% | ***** |
| Koskan et al., 2016 [6] | Yes | Yes | Yes | Yes | Yes | No | Can't tell | 60% | *** |
| Koskan et al., 2018 [7] | Yes | Yes | Yes | Yes | Yes | Yes | Yes | 100% | ***** |
| Kutner et al., 2021 [8] | Yes | Yes | Yes | Yes | Yes | Yes | Yes | 100% | ***** |
| Ong et al., 2014 [9] | Yes | Yes | Yes | Yes | Yes | Yes | Yes | 100% | ***** |
| Ong et al., 2015 [10] | Yes | Yes | Yes | Yes | Yes | Yes | Yes | 100% | ***** |
| Russo et al., 2018 [11] | Yes | Yes | Yes | Yes | Yes | Yes | No | 80% | **** |
| ***Quantitative randomized controlled trial*** | | | | | | | | | |
| Author and year | All Q1 | All Q2 | Is randomization appropriately performed? | Are the groups comparable at baseline? | Are there complete outcome data? | Are outcome assessors blinded to the intervention provided? | Did the participants adhere to the assigned intervention? | Quality | |
| Davis et al., 2013 [12] | Yes | Yes | Yes | Can't tell | Yes | Yes | no | 60% | *** |
| Nyitray et al., 2023 [13] | Yes | Yes | Yes | Yes | Yes | No | Yes | 80% | **** |
| ***Quantitative non-randomized studies*** | | | | | | | | | |
| Author and year | All Q1 | All Q2 | Are the participants representative of the target population? | Are measurements appropriate regarding both the outcome and intervention (or exposure)? | Are there complete outcome data? | Are the confounders accounted for in the design and analysis? | During the study period, is the intervention administered (or exposure occurred) as intended? | Quality | |
| Nyitray et al., 2018 [25] | Yes | Yes | Yes | Yes | Yes | Yes | Yes | 100% | ***** |
| Cruz et al., 2023 [15] | Yes | Yes | Can't tell | Can't tell | Yes | Yes | yes | 60% | *** |
| D'Souza et al., 2008 [17] | Yes | Yes | Yes | Can't tell | Yes | Yes | Yes | 80% | **** |
| Fenkl et al., 2015 [18] | Yes | Yes | Yes | Can't tell | Yes | Yes | Yes | 80% | **** |
| Fenkl et al., 2016 [19] | Yes | Yes | Yes | Yes | Yes | Yes | Yes | 100% | ***** |
| Gillis et al., 2022 [20] | Yes | yes | yes | Yes | Yes | Yes | Yes | 100% | ***** |
| Kutner et al., 2022 [21] | Yes | Yes | Yes | Yes | Yes | Yes | Yes | 100% | ***** |
| Li et al., 2009 [22] | Yes | Yes | Can't tell | Yes | Yes | Yes | Yes | 80% | **** |
| Lombardo et al., 2022 [23] | Yes | Yes | Yes | Can't tell | Yes | Yes | Yes | 80% | **** |
| Moores et al., 2015 [24] | yes | yes | yes | Can't tell | yes | No | Yes | 60% | *** |
| Olusanya et al., 2022 [26] | Yes | Yes | Yes | Yes | Yes | No | Yes | 80% | **** |
| Rahman et al., 2019 [27] | Yes | Yes | Yes | Yes | No | No | Yes | 60% | *** |
| Reed et al., 2010 [28] | Yes | Yes | Yes | Can't tell | Yes | Can't tell | Yes | 60% | *** |
| D' Souza et al., 2013 [16] | Yes | Yes | Yes | Yes | Yes | Yes | no | 80% | **** |
| Truesdale et al., 2010 [29] | Yes | Yes | Yes | Yes | No | No | Yes | 60% | *** |
| ***Quantitative descriptive studies*** | | | | | | | | | |
| Author and year | All Q1 | All Q2 | Is the sampling strategy relevant to address the research question? | Is the sample representative of the target population? | Are the measurements appropriate? | Is the risk of nonresponse bias low? | Is the statistical analysis appropriate to answer the research question? | Quality | |
| Apaydin et al., 2018b [14] | Yes | Yes | Yes | Yes | Yes | Can't tell | Yes | 80% | **** |
| ***Mixed method study*** | | | | | | | | | |
| Author and year | All Q1 | All Q2 | Is there an adequate rationale for using a mixed methods design to address the research question? | Are the different components of the study effectively integrated to answer the research question? | Are the outputs of the integration of qualitative and quantitative components adequately interpreted? | Are divergences and inconsistencies between quantitative and qualitative results adequately addressed? | Do the different components of the study adhere to the quality criteria of each tradition of the methods involved? | Quality | |
| Apaydin et al., 2018a [30] | Yes | Yes | No | Yes | Yes | Can't tell | No | 40% | ** |
| Ong et al., 2013 [32] | Yes | Yes | No | Yes | No | Can't tell | No | 20% | * |
| Hughes et al., 2022 [31] | yes | yes | No | Yes | No | Can't tell | No | 20% | * |

**Reference**

1. Acree ME, McNulty M, Blocker O, Schneider J, Williams HS. Shared decision-making around anal cancer screening among black bisexual and gay men in the USA. Cult Health Sex. 2020;22(2):201-16.

2. Butame SA, Lawler S, Hicks JT, Wilkerson JM, Hwang LY, Baraniuk S, et al. A qualitative investigation among men who have sex with men on the acceptability of performing a self- or partner anal exam to screen for anal cancer. Cancer Causes Control. 2017;28(10):1157-66.

3. Ejaz M, Ekström AM, Ahmed A, Haroon A, Ali D, Ali TS, et al. Human Papillomavirus associated prevention: knowledge, attitudes, and perceived risks among men who have sex with men and transgender women in Pakistan: a qualitative study. BMC Public Health. 2022;22(1):378.

4. Ejaz M, Ekström AM, Ali TS, Salazar M, Ahmed A, Ali D, et al. Integration of human papillomavirus associated anal cancer screening into HIV care and treatment program in Pakistan: perceptions of policymakers, managers, and care providers. BMC Public Health. 2023;23(1):1034.

5. Finneran C, Johnson Peretz J, Blemur D, Palefsky J, Flowers L. "That's Only for Women": The Importance of Educating HIV-Positive Sexual Minority Men on HPV and High Resolution Anoscopy (HRA). J Int Assoc Provid AIDS Care. 2021;20:23259582211016134.

6. Koskan AM, LeBlanc N, Rosa-Cunha I. Exploring the Perceptions of Anal Cancer Screening and Behaviors Among Gay and Bisexual Men Infected With HIV. Cancer Control. 2016;23(1):52-8.

7. Koskan AM, Fernandez-Pineda M. Anal Cancer Prevention Perspectives Among Foreign-Born Latino HIV-Infected Gay and Bisexual Men. Cancer Control. 2018;25(1):1073274818780368.

8. Kutner BA, Simoni JM, Aunon FM, Creegan E, Balán IC. How Stigma Toward Anal Sexuality Promotes Concealment and Impedes Health-Seeking Behavior in the U.S. Among Cisgender Men Who Have Sex with Men. Archives of Sexual Behavior. 2021;50(4):1651-63.

9. Ong JJ, Temple-Smith M, Chen M, Walker S, Grulich A, Fairley CK. Exploring anal self-examination as a means of screening for anal cancer in HIV positive men who have sex with men: a qualitative study. BMC Public Health. 2014;14:1257.

10. Ong JJ, Temple-Smith M, Chen M, Walker S, Grulich A, Hoy J, et al. Why are we not screening for anal cancer routinely - HIV physicians' perspectives on anal cancer and its screening in HIV-positive men who have sex with men: a qualitative study. BMC Public Health. 2015;15:67.

11. Russo S, McCaffery K, Ellard J, Poynten M, Prestage G, Templeton DJ, et al. Experience and psychological impact of anal cancer screening in gay, bisexual and other men who have sex with men: a qualitative study. Psychooncology. 2018;27(1):125-31.

12. Davis TW, Goldstone SE, Chen G. Tolerability of anal dysplasia screening. Journal of Lower Genital Tract Disease. 2013;17(4):404-8.

13. Nyitray AG, Nitkowski J, McAuliffe TL, Brzezinski B, Swartz MD, Fernandez ME, et al. Home-based self-sampling vs clinician sampling for anal precancer screening: The Prevent Anal Cancer Self-Swab Study. Int J Cancer. 2023;153(4):843-53.

14. Apaydin KZ, Fontenot HB, Shtasel DL, Mayer KH, Keuroghlian AS. Primary Care Provider Practices and Perceptions Regarding HPV Vaccination and Anal Cancer Screening at a Boston Community Health Center. J Community Health. 2018;43(4):792-801.

15. Cruz G, Ramos-Cartagena JM, Torres-Russe JL, Colón-López V, Ortiz-Ortiz KJ, Pericchi L, et al. Barriers and facilitators to anal cancer screening among people living with HIV in Puerto Rico. BMC Public Health. 2023;23(1):1940.

16. D'Souza G, Rajan SD, Bhatia R, Cranston RD, Plankey MW, Silvestre A, et al. Uptake and predictors of anal cancer screening in men who have sex with men. Am J Public Health. 2013;103(9):e88-95.

17. D'Souza G, Cook RL, Ostrow D, Johnson-Hill LM, Wiley D, Silvestre T. Anal cancer screening behaviors and intentions in men who have sex with men. Journal of General Internal Medicine. 2008;23(9):1452-7.

18. Fenkl EA, Schochet E, Jones SG, da Costa BR. Evaluation of an HPV/Anal Cancer Screening Awareness Program for HIV-infected Men Who Have Sex With Men. J Assoc Nurses AIDS Care. 2015;26(4):492-7.

19. Fenkl EA, Jones SG, Schochet E, Johnson P. HPV and Anal Cancer Knowledge among HIV-Infected and Non-Infected Men Who Have Sex With Men. LGBT Health. 2016;3(1):42-8.

20. Gillis JL, Grennan T, Grewal R, Ogilvie G, Gaspar M, Grace D, et al. Influence of previous experience with and beliefs regarding anal cancer screening on willingness to be screened among men living with HIV. BMC Public Health. 2022;22(1):2444.

21. Kutner BA, Simoni JM, DeWitt W, Gaisa MM, Sandfort TGM. Gay and bisexual men who report anal sex stigma alongside discomfort discussing anal sex with health workers are less likely to have ever received an anal examination or anal swab. LGBT health. 2022;9(2):103-13.

22. Li AH, Phanuphak N, Sahasrabuddhe VV, Chaithongwongwatthana S, Vermund SH, Jenkins CA, et al. Anal squamous intraepithelial lesions among HIV positive and HIV negative men who have sex with men in Thailand. Sex Transm Infect. 2009;85(7):503-7.

23. Lombardo J, Ko K, Shimada A, Nelson N, Wright C, Chen J, et al. Perceptions of and barriers to cancer screening by the sexual and gender minority community: a glimpse into the health care disparity. Cancer Causes Control. 2022;33(4):559-82.

24. Moores A, Phillips JC, O'Byrne P, MacPherson P. Anal cancer screening knowledge, attitudes, and experiences among men who have sex with men in Ottawa, Ontario. Canadian Journal of Human Sexuality. 2015;24(3):228-36.

25. Nyitray AG, Hicks JT, Hwang LY, Baraniuk S, White M, Millas S, et al. A phase II clinical study to assess the feasibility of self and partner anal examinations to detect anal canal abnormalities including anal cancer. Sex Transm Infect. 2018;94(2):124-30.

26. Olusanya OA, Tomar A, Thomas J, Johnson P, Wigfall LT. HPV-Associated Anal Cancer Knowledge, Attitudes, and Health Communication Behaviors Among Non-clinical Providers at HIV/AIDS Service Organizations in Southern United States Region. Journal of Cancer Education. 2022;37(6):1886-92.

27. Rahman M, Li DH, Moskowitz DA. Comparing the Healthcare Utilization and Engagement in a Sample of Transgender and Cisgender Bisexual+ Persons. Arch Sex Behav. 2019;48(1):255-60.

28. Reed AC, Reiter PL, Smith JS, Palefsky JM, Brewer NT. Gay and bisexual men's willingness to receive anal Papanicolaou testing. Am J Public Health. 2010;100(6):1123-9.

29. Truesdale MD, Goldstone SE. The fear factor: drivers and barriers to follow-up screening for human papillomavirus-related anal cancer in men who have sex with men. Int J STD AIDS. 2010;21(7):482-8.

30. Apaydin KZ, Nguyen A, Panther L, Shtasel DL, Dale SK, Borba CPC, et al. Facilitators of and barriers to high-resolution anoscopy adherence among men who have sex with men: a qualitative study. Sex Health. 2018;15(5):431-40.

31. Hughes R, Fitzpatrick C, Nichols K, Devlin J, Richardson D. A pilot study exploring sexual health clinician confidence and barriers to anal examination and proctoscopy in men who have sex with men. Sex Health. 2022;18(6):515-6.

32. Ong J, Chen M, Temple-Smith M, Walker S, Hoy J, Woolley I, et al. The inside story. Physicians' views on digital ano-rectal examination for anal cancer screening of HIV positive men who have sex with men. J Med Screen. 2013;20(4):188-91.
